# The ‘onion skin’ sign of a low‐grade appendiceal mucinous neoplasm: An incidental finding during early pregnancy assessment

**DOI:** 10.1002/ajum.12377

**Published:** 2024-03-12

**Authors:** Hanine Fourie, Maya Al Memar, Maeve Tuomey, Catriona Stalder, Paul Ziprin, Tom Bourne

**Affiliations:** ^1^ Tommy's National Centre for Miscarriage Research Imperial College NHS Trust, Queen Charlotte and Chelsea Hospital London UK; ^2^ Department of Colorectal Surgery Imperial College NHS Trust, St Mary's Hospital London UK; ^3^ Department of Development and Regeneration KU Leuven Leuven Belgium

**Keywords:** appendiceal mucocele, case report, early pregnancy, incidental findings

## Abstract

A low‐grade appendiceal mucinous neoplasm (LAMN) is a cystic dilatation of the appendix resulting from the accumulation of mucinous secretions caused by a luminal obstruction. Although usually benign, pseudomyxoma peritonei may occur in the event of rupture, and 10% of cases may be secondary to appendiceal cystadenocarcinoma. A LAMN is both more common and more likely to have a malignant association in women, making it an entity with which practitioners of gynaecological ultrasound should be familiar. Although not the primary aim, early pregnancy ultrasound assessments can offer the diagnostic opportunity to identify pelvic pathology. A LAMN can be identified on ultrasonography by visualisation of an adnexal mass separate to the ovary, which due to the layers of secretions has a distinctive appearance previously likened to ‘onion‐skin’ or ‘whipped‐cream’. Here, we describe an incidental finding of a LAMN during an early pregnancy assessment. Practitioners of early pregnancy ultrasound should be familiar with the characteristic morphology of this rare but important finding.

## Introduction

Ultrasound scans are performed in early pregnancy to confirm location, viability, and the presence or absence of multiple pregnancy. It may also identify incidental pelvic pathology such as ovarian masses and uterine lesions. Some of these findings may carry risk to the mother, with a reported 3% incidence of an ovarian cyst accident such as torsion in a large case series.^1^ Therefore, not only does a decision need to be made about whether to treat but, given the context of pregnancy, when to treat.

A low‐grade appendiceal mucinous neoplasm (LAMN) can be mistakenly classified as an ovarian mass, most commonly a dermoid due to acoustic shadowing often being seen with both types of pathology, or as a para‐ovarian mass.^2^ A mass in the right lower quadrant, separate from the right ovary, should raise suspicion of a LAMN.^3^ Although the appearance of a mucocele on ultrasound is varied, a unilocular mass with layered hyperechoic content with acoustic shadows secondary to mural calcification may be present. This has been described as the specific ‘onion‐skin’ sign.^4^ Although rarely diagnosed by gynaecologists, Van Holsbeke *et al*. proposed the ‘whipped‐cream’ sign to describe its morphology.^5^

Studies testing the accuracy of these characteristic signs rely on the retrospective review of ultrasound images in which the diagnosis is already known. Our case study describes an ultrasonographer making a correct diagnosis of LAMN prospectively based on prior knowledge of the characteristic ‘onion‐skin’ sign.

## Case summary (patient information, clinical findings, diagnostic assessment, therapeutic intervention and follow‐up)

**Figure 1:**
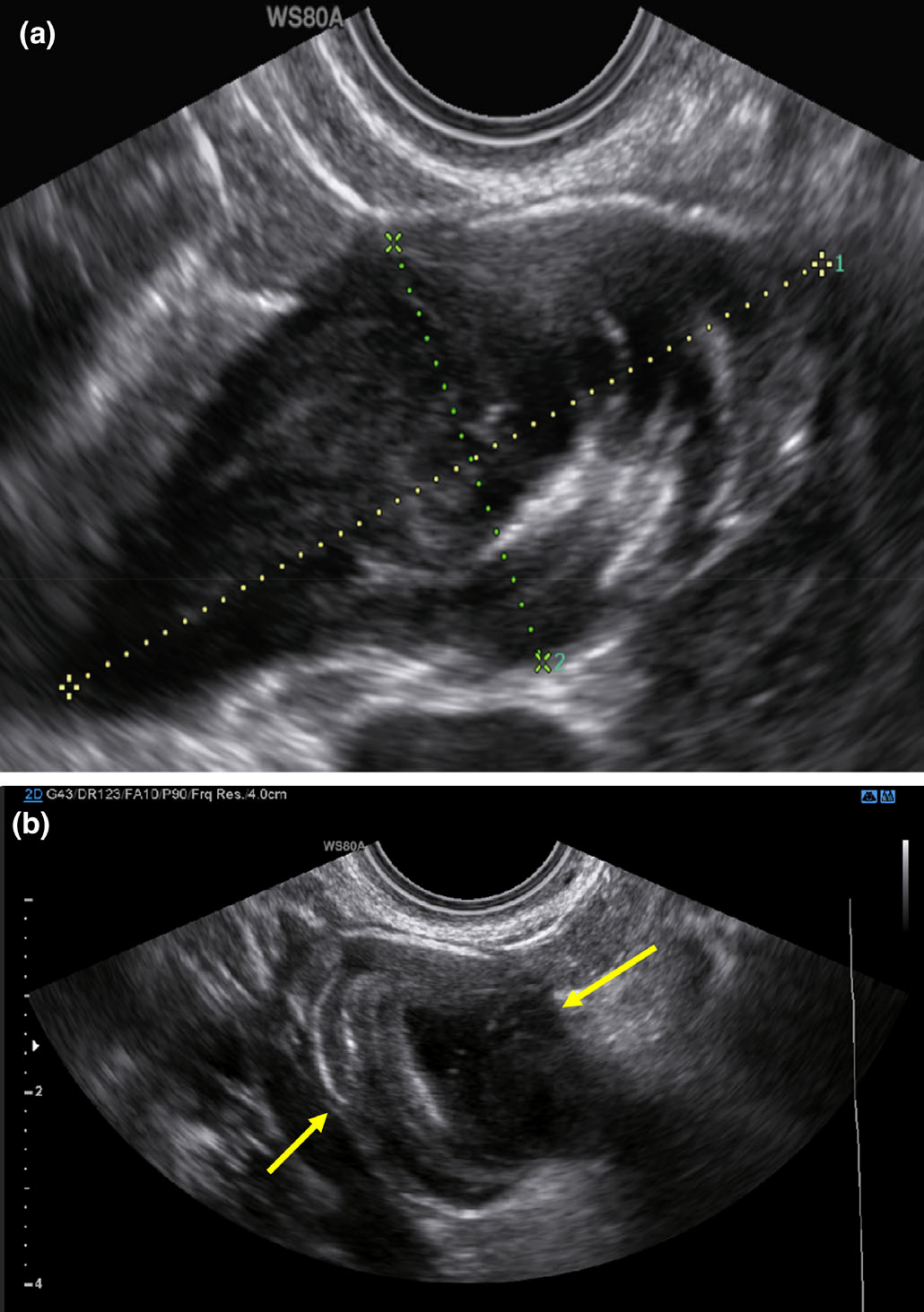
Transvaginal ultrasound scan showing hyperechoic concentric lines representing mucin deposition of the low grade appendiceal mucinous neoplasm (LAMN) at 6 weeks of pregnancy. The mass measured 60 millimetres (a) by 30 millimetres (b).

A 37‐year‐old pregnant woman presented to our unit at five weeks of gestation with a history of bleeding. The pregnancy was classified as a pregnancy of unknown location; however, a 3‐cm adnexal mass was noted on ultrasonography, which was initially thought to be a dermoid cyst. An ultrasound scan one week later confirmed an intrauterine pregnancy, and the adnexal mass was shown to be separate from the ovary (Figure 1). The experienced operator recognised the unilocular appearance of the mass with its concentric hyperechogenic lines as a LAMN.

**Figure 2:**
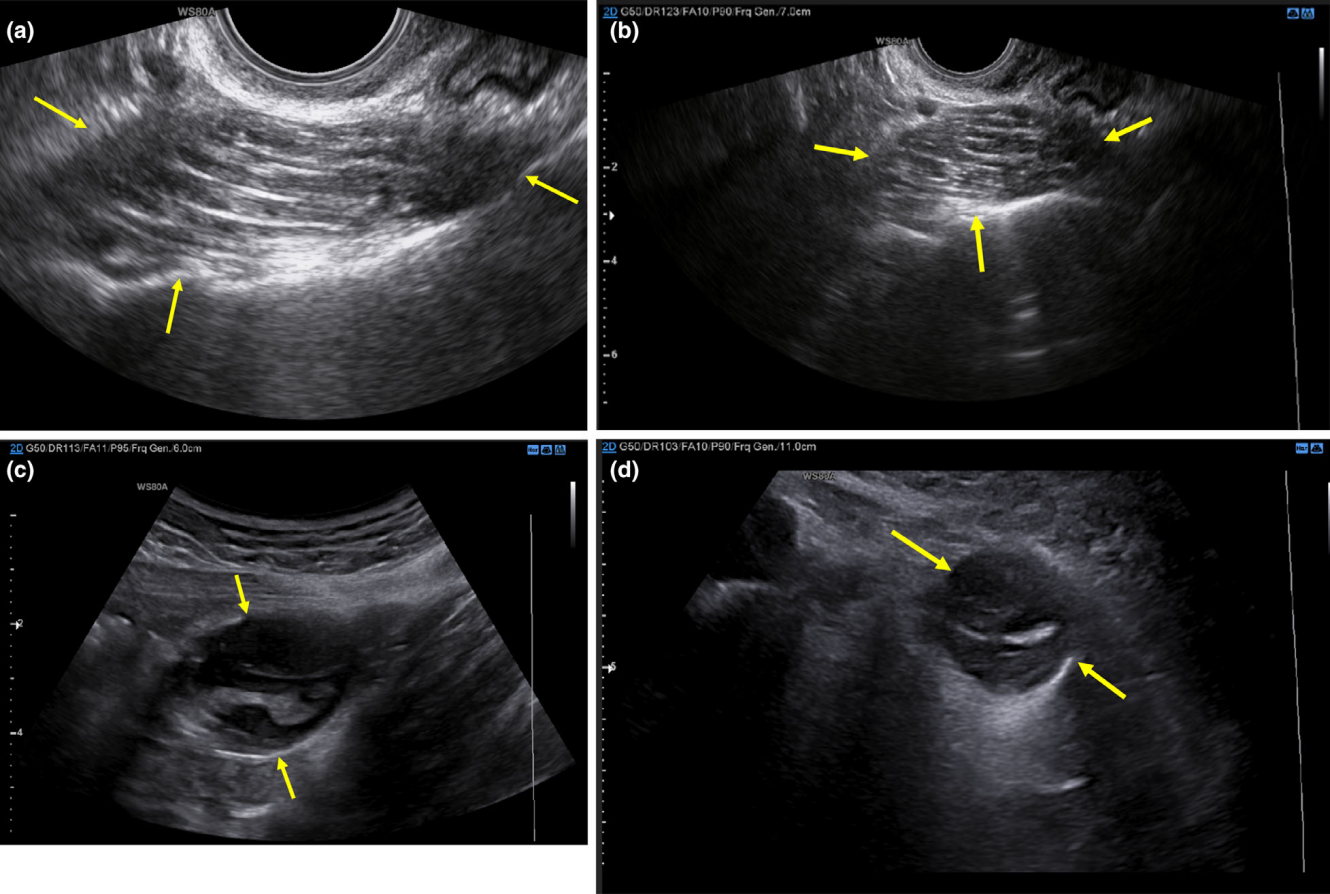
(a) Transvaginal ultrasound scan showing the appearance of the low‐grade appendiceal mucinous neoplasm at 8 weeks of pregnancy. (b) Transvaginal ultrasound scan in transverse showing the appearance of the low‐grade appendiceal mucinous neoplasm at 8 weeks of pregnancy. (c) Transabdominal (TA) ultrasound scan showing the appearance of the low‐grade appendiceal mucinous neoplasm (LAMN) at 8 weeks of pregnancy. The lower resolution from the lower frequency of TA shows a different appearance of the LAMN compared with the transvaginal images. (d) Transabdominal ultrasound scan in transverse section showing the appearance of the low‐grade appendiceal mucinous neoplasm at 8 weeks of pregnancy.

**Figure 3:**
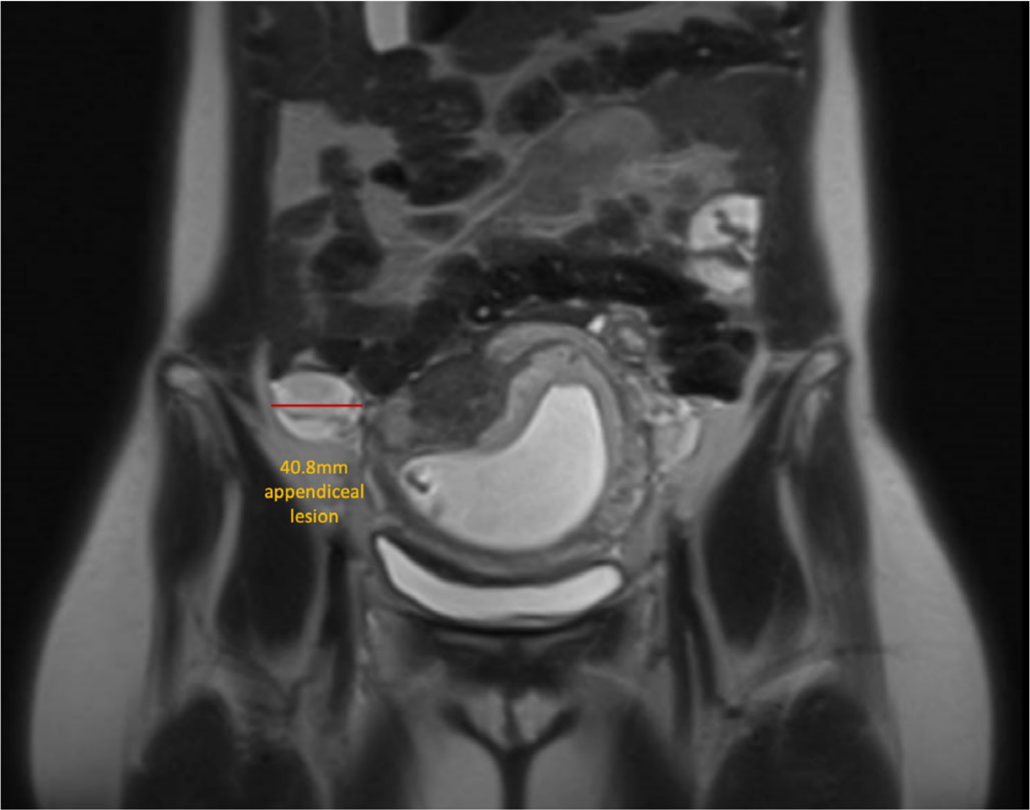
T2‐weighted MRI of pelvis at 13/40 weeks of pregnancy illustrating the gravid uterus with the 40.8‐mm hyperintensity appendiceal lesion to the right.

She was referred to the surgical team at 13 weeks' gestation (Figure 2). They arranged pelvic magnetic resonance imaging, which supported the diagnosis and did not identify the involvement of the caecum (Figure 3). Since she was asymptomatic and pregnant, a plan was made for a postpartum appendicectomy.

**Figure 4:**
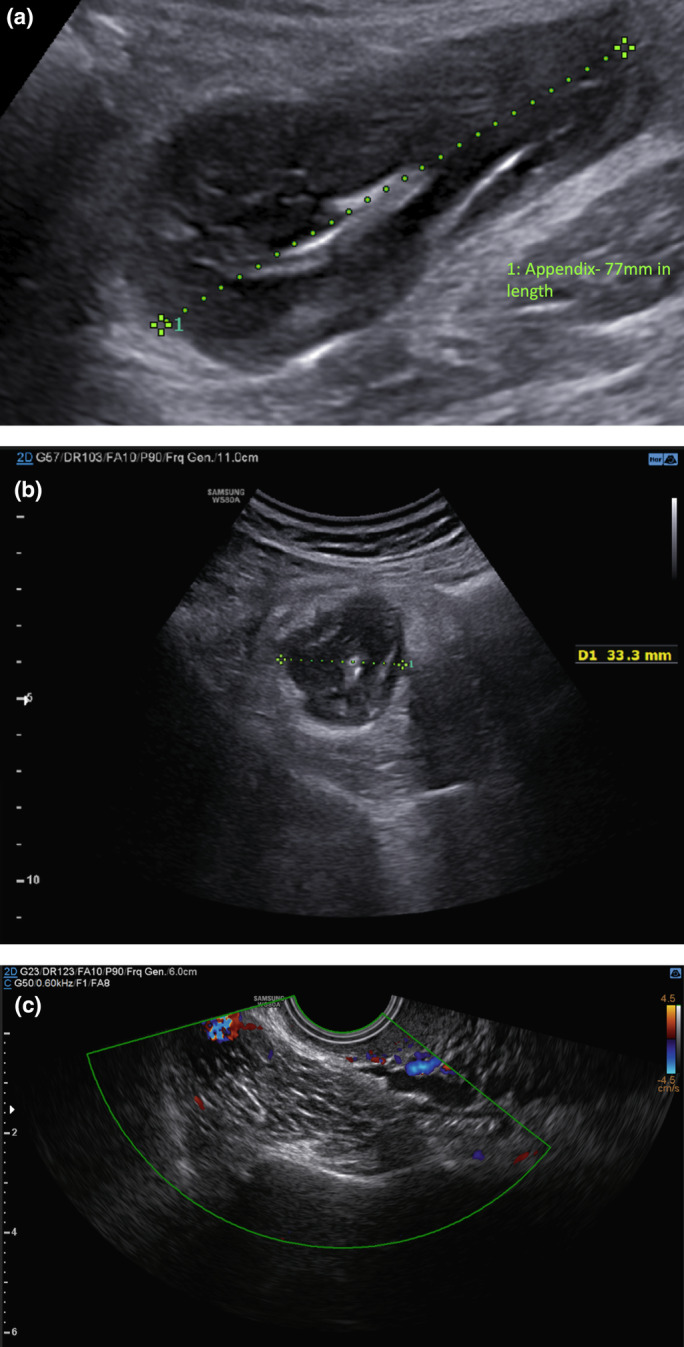
(a) Transabdominal ultrasound image of the low‐grade appendiceal mucinous neoplasm 10 weeks of post‐vaginal birth measuring 77 mm in length. (b) Transabdominal ultrasound image in transverse section of the low‐grade appendiceal mucinous neoplasm 10 weeks of post‐vaginal birth measuring 33.3 mm in width. (c) Transvaginal ultrasound image of the low‐grade appendiceal mucinous neoplasm 10 weeks of post‐vaginal birth.

**Figure 5:**
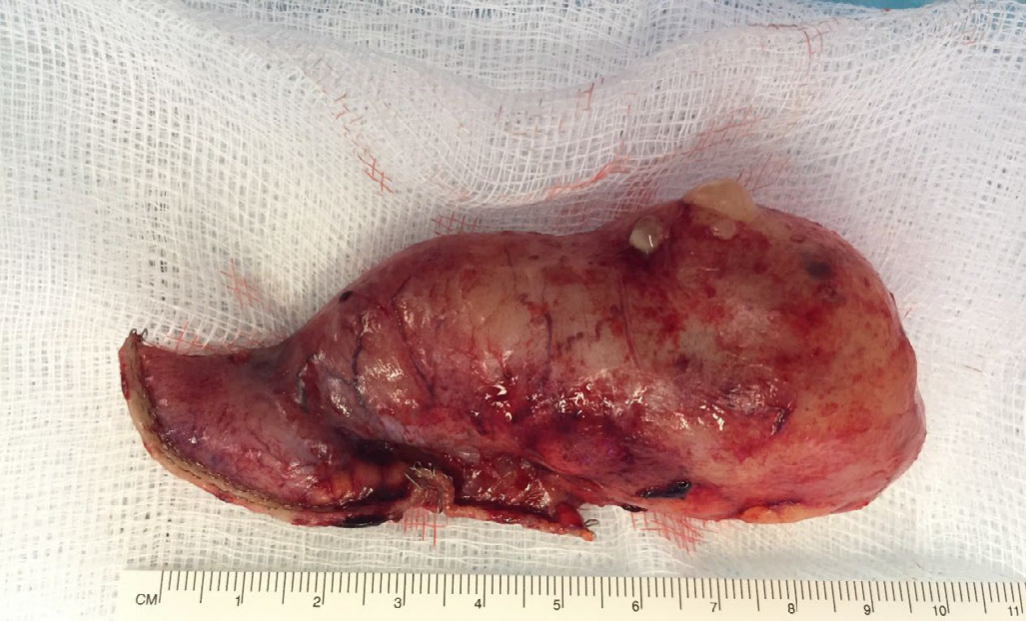
Surgical specimen of the low‐grade appendiceal mucinous neoplasm—a dilated appendix measuring 90 mm in length and approximately 40 mm across the base. Focally, there is a clear ‘bleb’ on the serosa with some possible exudate suggestive of perforation.

Ten weeks after a vaginal delivery, she returned to clinic with a 4‐day history of right iliac fossa pain prior to her planned review (Figure 4). A laparoscopic appendicectomy was performed with no immediate evidence of appendiceal perforation or free fluid. The histopathology of the appendix confirmed the diagnosis of a LAMN with low‐grade dysplastic epithelium. There was a small perforation site with marked serositis and calcification of the appendiceal wall (Figure 5). Given the perforation site on histopathology, the patient was followed‐up over five years with MRI pelvis, colonoscopy and tumour markers. She has now been discharged.

## Discussion

The reported incidence of LAMN is based on identifying the pathology in surgical specimens, which are usually removed when patients present with symptoms acutely. The prevalence of silent LAMN is not known. In some series, up to 36% of LAMN are secondary to a cystadenocarcinoma,^6^ and even if benign, rupture can lead to malignant pseudomyxoma peritonei. A presentation with abdominal pain, weight loss, an abdominal mass and a mass size >2 cm are more readily associated with malignancy.^6^

If the diagnosis is not made incidentally during routine ultrasonography, it may be made during acute cases of appendicitis or peritonitis in pregnancy,^7^ or intra‐operatively during a gynaecological procedure to remove a suspected adnexal mass.^8^ In the largest surgical case series of LAMN (135 cases over 24 years), only 19% of patients had an accurate pre‐operative diagnosis, with only two being identified on ultrasonography.^6^ Pre‐operative planning and specialist removal ensure curative surgery and a reduced risk of intraoperative spread.

A LAMN can be identified by imaging. As described, the ‘onion skin’ sign on ultrasonography had a reported specificity of 100% and sensitivity of 63% for the diagnosis of LAMN in a small study reviewing ultrasound images of colorectal pathologies, which included five LAMNs.^9^ Computed tomography (CT) of the abdomen can be used to identify LAMN if the appendiceal lumen is more than 1.3 cm and if cystic dilatation and mural calcification are present. Computed tomography can also help differentiate between benign and malignant causes.^10^ However, this modality is contraindicated in pregnancy, whereas MRI is thought to be comparatively safe. MRI has similar diagnostic accuracy to CT, but mural calcifications are less apparent.^11^ Colonoscopy can also identify the pathology. Classically this is evident as either a smooth indentation of the caecal lumen or a mass arising from the appendiceal orifice that moves with breathing.

Surgical resection of the appendix is usually curative. A right hemicolectomy is often performed for mucinous cystadenocarcinoma, whereas for benign pathologies such as hyperplasia and cystadenoma, appendicectomy is sufficient. Conversion to an open procedure during laparoscopy is recommended if spillage of the content is suspected.

Sonographers responsible for early pregnancy scans should be familiar with the onion skin sign as the specific appearance of an LAMN to facilitate prompt referral to the appropriate specialist team, and to prevent complications such as pseudomyxoma peritonei.

## Conclusion

Recognition of incidental pelvic pathology during early pregnancy ultrasonography is important. Here, we describe a relatively rare condition that sonographers may encounter. Being familiar with the characteristic features will raise diagnostic suspicion and guide appropriate referral and management.

### Patient perspective

The patient supported this publication. She wishes to increase awareness and our ability to diagnose LAMN. She explained that the antenatal diagnosis of an appendiceal mucocele motivated her to present promptly when she experienced pelvic pain after delivery. She said that without this knowledge, she may have disregarded her mild symptoms. It was only at surgical follow‐up that she fully appreciated the benefit of early diagnosis and treatment in preventing possible disseminated malignancy.

### Patient consent

The patient provided written informed consent for a prospective cohort study, and specifically for this case report.

### Authorship statement

Conceptualisation: HF, MAM, TB. Funding acquisition: HF, MAM, TB. Investigation: HF, MAM, CS, MT, PZ, TB. Supervision: PZ, TB. Writing ‐ draft: HF. Writing‐ review and editing: HF, MAM, CS, MT, PZ, TB.

## Funding

No funding information is provided.

## Conflict of interest

None to declare.

## Ethics statement

The patient was enrolled in the EPOS2 prospective cohort, which was approved by NHS National Research Ethics Service (NRES) NHS North East—Newcastle and North Tyneside Research Ethics Committee (17/NE/0121). She provided written informed consent and additional consent for this case report.
